# The relationship between DNA methylation, genetic and expression inter-individual variation in untransformed human fibroblasts

**DOI:** 10.1186/gb-2014-15-2-r37

**Published:** 2014-02-20

**Authors:** James R Wagner, Stephan Busche, Bing Ge, Tony Kwan, Tomi Pastinen, Mathieu Blanchette

**Affiliations:** 1 Department of Human Genetics, McGill University, 740 Dr. Penfield Avenue, Room 6202, Montréal, Québec H3A 0G1, Canada; 2 Génome Québec Innovation Centre, 740 Dr. Penfield Avenue, Room 6202, Montréal, Québec H3A 0G1, Canada; 3 McGill University School of Computer Science, Rm 318, 3480 University Street, Montreal, Québec H3A 0E9, Canada

## Abstract

**Background:**

DNA methylation plays an essential role in the regulation of gene expression. While its presence near the transcription start site of a gene has been associated with reduced expression, the variation in methylation levels across individuals, its environmental or genetic causes, and its association with gene expression remain poorly understood.

**Results:**

We report the joint analysis of sequence variants, gene expression and DNA methylation in primary fibroblast samples derived from a set of 62 unrelated individuals. Approximately 2% of the most variable CpG sites are mappable in *cis* to sequence variation, usually within 5 kb. Via eQTL analysis with microarray data combined with mapping of allelic expression regions, we obtained a set of 2,770 regions mappable in *cis* to sequence variation. In 9.5% of these expressed regions, an associated SNP was also a methylation QTL. Methylation and gene expression are often correlated without direct discernible involvement of sequence variation, but not always in the expected direction of negative for promoter CpGs and positive for gene-body CpGs. Population-level correlation between methylation and expression is strongest in a subset of developmentally significant genes, including all four *HOX* clusters. The presence and sign of this correlation are best predicted using specific chromatin marks rather than position of the CpG site with respect to the gene.

**Conclusions:**

Our results indicate a wide variety of relationships between gene expression, DNA methylation and sequence variation in untransformed adult human fibroblasts, with considerable involvement of chromatin features and some discernible involvement of sequence variation.

## Background

Perhaps the best studied of epigenetic phenomena, the methylation of CpG dinucleotides, has been known for many years to play a key role in X-chromosome inactivation [1], transcriptional silencing of foreign DNA elements [2] and imprinting of genes [3], while aberrant DNA methylation is implicated in many types of cancer [4]. The relationship between methylation and gene expression is complex, with high levels of gene expression often associated with low promoter methylation [5] but elevated gene body methylation [6], and the causality relationships have not yet been determined. In cell populations, the levels of DNA methylation across CpG sites in the genome is typically regarded as bimodal, with CpG-rich regions known as CpG islands, often associated with transcription start sites (TSSs), typically showing hypomethylation, and other CpG sites showing hypermethylation (reviewed in [7]).

Methylation has been shown to be highly variable across cell types with variable sites falling in two broad categories: those with inverse correlation between DNA methylation and chromatin accessibility, and those with variable chromatin accessibility and constitutive DNA hypomethylation [8]. As reviewed by Cedar and Bergman [9], DNA methylation and histone modifications share many relationships from the time of embryonic development onwards, including hypothesized roles of DNA methylation preventing the tri-methylation of histone 3 lysine 4 (H3K4me3), a marker generally associated with active promoters, as well as H3K4me3 preventing DNA methylation [10].

Methylation also varies between healthy individuals in a population. Relationships between DNA methylation, gene expression and various other genetic and epigenetic biomarkers have been examined previously. Recent studies have identified SNPs whose genotype correlates with DNA methylation (termed methylation quantitative trait loci, or mQTLs) in various human populations and cell types. Bell *et al*. [11] utilized the HumanMethylation27 BeadChips from Illumina to map associations between SNPs and methylation levels at 22,290 CpG dinucleotides in lymphoblastoid cell lines (LCLs), finding 180 CpG sites associated with nearby SNPs, and an enrichment for expression QTLs (eQTLS) amongst mQTLs. Gibbs *et al*. [12] used the same DNA methylation platform to study samples from four human brain regions in 150 individuals and reported hundreds of SNP-associated CpG sites in each brain tissue, with mQTLs typically located very close to the associated CpG site, and thousands of both mQTLs and eQTLs, but only modest overlaps between the two, averaging 13 CpG sites per tissue having a significant mQTL that was also an eQTL. Similar results were seen using 180 LCLs derived from one African and one European population [13]. Zhang *et al.*[14] performed similar analyses using the same methylation platform in 153 human adult cerebellum samples, finding 2,046 CpG sites with mQTLs; they reported that, in general, CpG sites located in CpG islands are more likely to be mappable to a SNP than non-CpG island sites. They also assessed the relationship between expression and methylation, with 20 of 112 CpG-gene pairs analyzed showing nominally significant correlations, with 5 of these 20 being positive correlations and the rest negative. At present, though it is known that there is a genetic component to both variable DNA methylation and gene expression, as well as genome-level differences in gene expression linked to DNA methylation, the combined relationships between the three factors remains poorly understood. Recent research [15] has examined the relationship between sequence, expression and DNA methylation as measured by the HumanMethylation27 assay in whole blood, finding numerous cases of methylation/expression relationships but focusing on the small number of cases in which a genetic component was also found. Drong *et al.*[16] report 149 CpG sites mappable to an mQTL when making use of differential methylation hybridization covering 27,718 genomic regions in 38 unrelated individuals, finding none of the mQTLs to also be eQTLs. Gutierrez-Arcelus *et al.*[17] report positive and negative expression-methylation relationships at the inter-individual level in fibroblasts, T cells and LCLs derived from a set of 204 umbilical cords from healthy newborns of European descent, with negatively correlated CpG sites enriched at ENCODE derived enhancer and promoter sites.

To further understand the relationship between genetics, gene expression, DNA methylation, and other epigenetic marks, we present analyses of DNA methylation, gene expression (both total and allelic) and DNA sequence polymorphisms from a set of 62 fibroblast cell lines derived from healthy human individuals, augmented with publicly available histone mark and DNase I hypersensitivity (DHS) data. We show that: widespread relationships exist between DNA polymorphisms and DNA methylation (mQTLs); widespread relationships exist between DNA methylation and gene expression, especially in developmentally significant genes, including all four HOX clusters; supplementing expression quantitative trait locus (eQTL) data with mapping of allelic expression to adjacent SNPs, a large set of regions and genes mapped to a QTL that also functions as an mQTL, comprising 242 genes and 23 regions not overlapping with an annotated gene; and CpG sites where methylation correlates with gene expression in *cis* do not, in general, show strong overlap with annotated genes or promoter regions - rather, CpG sites where this correlation is negative are most commonly seen in sites associated with active promoter marker H3K4me3 and DHS regions, while those with positive correlation are most commonly seen in the presence of the repressive chromatin marker H3K27me3 (histone 3 lysine 27 tri-methylation).

## Results

We report on the joint analysis of inter-individual variation in the levels of DNA methylation, total and allelic expression, and DNA sequence of 62 healthy parents of 31 parent-child trios of European descent. Here, we start by introducing each data set individually before discussing the relationships among them.

### DNA methylation assays

DNA methylation was assayed in forearm skin fibroblast samples using the Illumina 450 K assay (Materials and methods). For each sample, methylation was measured at approximately 485,000 CpG sites, but we only considered the approximately 392,000 sites uniquely mapped in autosomes and containing no known SNPs. Methylation levels are measured in populations of diploid cells using beta values [18], which range from 0 (no methylation) to 1 (complete methylation of the two alleles). Methylation measurements were highly replicable, with the Pearson correlation coefficient between beta values of two replicates exceeding 0.99 in each of three pairs of biological replicates, while the average pairwise correlation coefficient between methylation from different samples levels ranges around 0.95; Additional file 1). Surrogate Variable Analysis [19] was used to identify possible batch effects accounting for inter-individual methylation variation but none were detected, suggesting that the observed variation may mostly be due to stochastic, environmental, or genetic effects.

**Figure 1:**
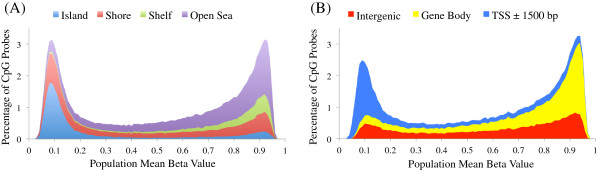
**Fibroblast methylation beta values are bimodal and the two modes show different breakdown in terms of CpG islands and genes.** Distribution of methylation beta values in type II probes across the genome, partitioned by position relative to **(A)** CpG islands (with a shore defined by Illumina as less than 2 kb from an annotated CpG island, a shelf as 2 to 4 kb, and open sea as more than 4 kb) and **(B)** annotated genes.

The Illumina 450 K assay includes both type I probes utilizing two query probes per CpG locus (largely concentrated around genes’ TSSs), and type II probes utilizing a single probe per locus (dispersed somewhat more uniformly across the genome; see Materials and methods). The distributions of methylation beta values differ for type I and type II probes due to their localization biases but both are bimodal, with modes corresponding to CpG sites that are unmethylated in most cells of the sample (hypomethylated), and those that are methylated in most cells of the sample (hypermethylated) (Figure 1A (type II probes); Additional file 2A (type I probes)). Consistent with previous reports [7], hypomethylated sites are mainly located in CpG islands and within 1.5 kb of the TSS of a gene (53% of probes with mean beta value <0.3 are located near a TSS versus 34% of all probes; in the case of CpG islands, it is 60% versus 32%), whereas hypermethylated sites are generally located in the rest of the genome (distal intergenic and gene body regions).

**Figure 2:**
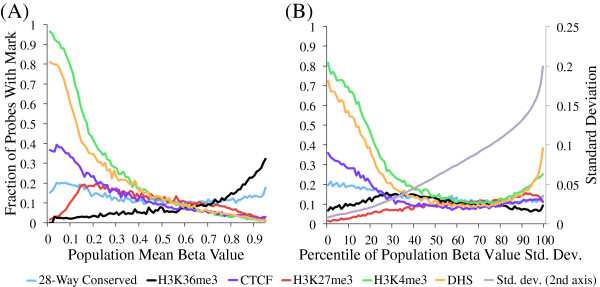
**Mean and variance of beta values of CpG probes associate with several genome marks.** Proportion of type II CpG probes falling in various types of genomics regions identified by ENCODE, partitioned by **(A)** CpG probe mean beta value and **(B)** percentile of beta value standard deviation (Std. dev.). All data types, except for 28-way conservation, are derived from broad peaks in BJ human foreskin fibroblast cells.

**Figure 3:**
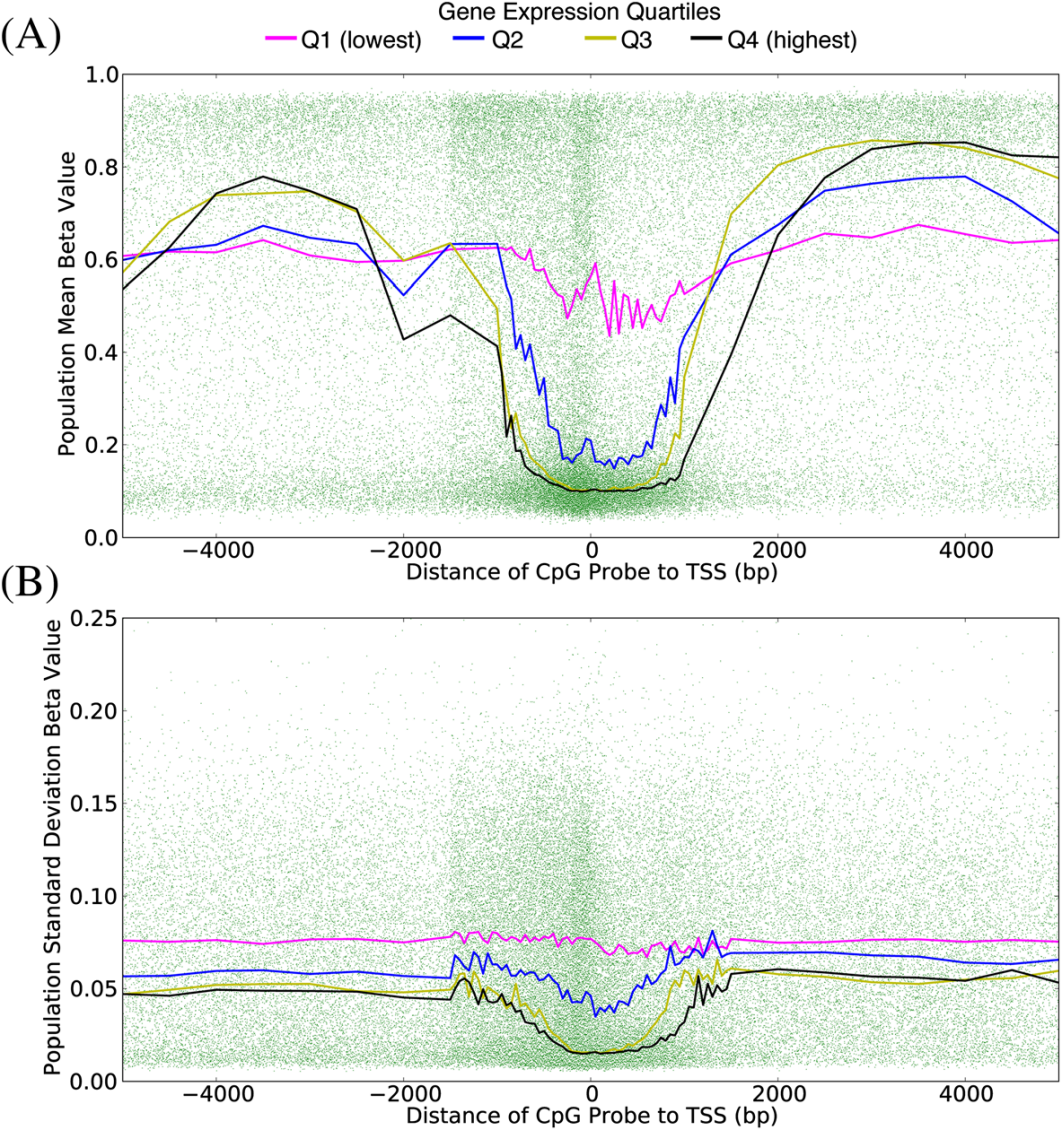
**The mean and variance of beta values of CpG probes near transcription start sites depend on the gene’s expression level.** Mean **(A)** and standard deviation **(B)** of type II CpG probes with respect to their position relative to TSSs of annotated genes. Each green dot corresponds to a CpG probe, and the four lines show the running median for probes based on the quartile of the expression level (from RNA-seq in four individuals) of the gene they are associated with.

Hypomethylated CpG sites are preferentially located in active regulatory regions characterized by DHS and H3K4me3, as measured by the ENCODE consortium in fibroblast cell lines [20] (Figure 2A (type II probes); Additional file 3A (type I probes)). Of hypomethylated CpG sites, 59% overlap with a DHS peak in the BJ foreskin fibroblast line, and 72% with an H3K4me3 peak. This is approximately twice the fraction seen among all CpG sites (29% and 34%, respectively). On the contrary, hypermethylated sites show a considerable overlap with H3K36me3, an intragenic marker of active transcription [21], with 19% of sites with mean beta >0.7 overlapping with a peak for this mark, compared to 9% among all sites. However, 62% of hypermethylated sites overlap none of the features considered in our analyses. Consistent with observations of low methylation in regions of DHS and active histone marks, genes with high expression levels show considerably lower methylation in the region proximal to the TSS (up to 1,500 bp from the TSS) and higher methylation in the gene body region compared to genes with lower average expression levels (Figure 3A; Additional file 4A), with probes adjacent to genes in the top quartile of expression having mean beta <0.3 81% of the time and mean beta >0.7 only 11%. Those in the lowest quartile still have a plurality of hypomethylated probes near the TSS, but with numbers considerably diminished, that is, 42% hypomethylated versus 30% hypermethylated.

We examined the levels of inter-individual variation of methylation probes, finding a drop in variation of probes located within 1,500 bp of a TSS annotated for an actively expressed gene (Figure 3B; Additional file 4B), with only 11% of probes near the TSS of a top quartile expression gene also being in the top quartile of methylation variation, compared to 30% for CpG sites adjacent to the TSS of a bottom quartile expression gene. These results were corroborated by the finding that sites with low inter-individual methylation variation were enriched for DHS and H3K4me3, and, to a lesser degree, sequence conservation (Figure 2B; Additional file 3B).

On the contrary, highly variable CpG probes (top 25%, standard deviation >0.0932) are usually located far away from the TSS (either in intergenic regions or in the gene body), or are located near the TSS of genes with low expression in fibroblasts and generally lack regulatory or evolutionary marks of function. The majority of these CpG sites show a unimodal distribution (Additional file 5). Genes whose TSS regions contain highly variable CpG probes were enriched for Gene Ontology (GO) terms related to multicellular organismal development (Additional file 6, worksheet 1), compared to the full set of genes having at least one CpG probe in the TSS region. Unexpectedly, extremely variable CpG probes (top 5%, standard deviation >0.15) show a marked increase in their overlap with DHS and H3K4me3 marks. Genes collocated with these CpG probes are even more strongly enriched for having functions related to development, and include a large number of genes from the HOX clusters (see Discussion).

### Gene expression analysis

RNA expression levels for the 62 individuals were measured using the Illumina HumanRef8 microarray platform, giving expression levels for 21,916 probes mapping to a total of 16,952 genes. Only probes that showed moderate to high inter-individual expression variation (standard deviation >0.1127, corresponding to a total of 9,493 genes) were considered for further analyses.

To complement total expression data, allelic expression (AE) was assayed at a set of approximately 900,000 SNP locations dispersed in annotated genes and intergenic regions of all autosomes using hybridization to genotyping arrays, as previously described [22] (see Materials and methods). For each sample and each heterozygous SNP, the ratio of the expression level of each allele is estimated, after normalization to genomic DNA. Of 24,814 known canonical UCSC genes, 81% have at least one assayed SNP within their boundaries. A previously described [23] hidden Markov model was used to reduce the noise in the data and estimate, for each SNP of each sample, the expected true allele expression log-ratio. We note that because this approach does not make use of gene annotation, it is able to detect AE at transcripts that do not, or only partially, overlap annotated genes. However, detection power for genes that are short or contain a small number of SNPs is reduced.

As previously reported for other cell types [22], AE was seen to be widespread. We defined an aeSNP as a SNP whose expected log_2_ allele ratio is above 0.2 in at least two samples (which corresponds to 5% false discovery rate (FDR); Materials and methods), and found 74,624 aeSNPs within annotated gene regions (corresponding to 15.8% of genic/intronic SNPs), and 25,467 outside (corresponding to 5.4% of intergenic SNPs). aeSNPs were clustered into 3,327 aeRegions (consisting of two or more consecutive aeSNPs), of which more than 80% had full or partial overlap with an annotated gene (Additional file 7), similar to results previously obtained in lymphoblasts [23] (for a full list of aeRegions, see Additional file 8).

### Linking methylation and genetic variation

Inter-individual methylation variation is likely due to both genetic and environmental variation between samples. To determine the relationship between genetic variation and CpG methylation levels, we first genotyped our 62 samples (Materials and methods). We then mapped CpG beta values to the imputed genotype at polymorphic sites within 250 kb (absolute value Spearman’s rho above 0.452, which corresponds to a *P*-value of 6 × 10^-6^ and an FDR of 5% (Materials and methods)). A set of 27,486 pairs (Additional file 9) were retained as significant, involving a total of 1,676 mappable CpG probes and 19,561 candidate mQTLs. Whole genome bisulfite sequencing-derived DNA methylation data were generated for four fibroblast cell lines (Additional file 10) and used to validate array methylation detected at mappable CpG loci. We observe high concordance between array- and sequencing-derived methylation for highly variable CpG sites across the four cell lines (254 loci; median Pearson correlation coefficient = 0.84).

**Figure 4:**
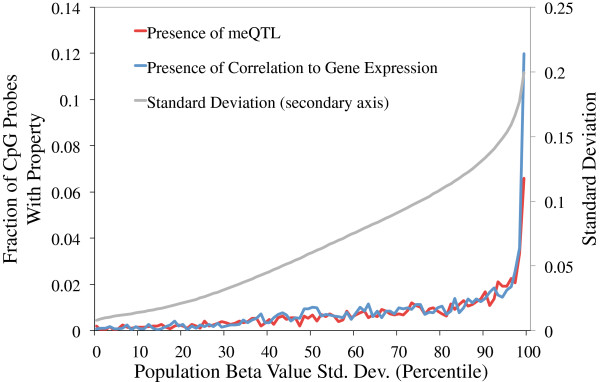
**Variable CpG sites are more likely to be correlated with expression or sequence.** Proportion of probes being significantly correlated (5% FDR) to either an mQTL or a gene’s expression levels, by percentile of population standard deviation.

Remarkably, mappable CpG probes are 1.5-fold enriched in fibroblast DHS regions, but 1.75-fold depleted in highly conserved regions. While CpG probes found within CpG islands are underrepresented in the set of highly variable CpG probes (Figure 1B), CpG island probes are 1.66-fold enriched in mappable probes when compared to the set of highly variable CpG probes. Although mappable CpG probes represent only 1.7% of all highly variable CpG probes, they are approximately four times more frequent among extremely variable CpG probes relative to the set of highly variable probes (Figure 4). Most mappable CpG probes have a distribution of methylation levels that is unimodal, consistent with a moderate effect of genetic variation on methylation. However, bimodality and trimodality are much more frequent among this set of CpG probes than in highly variable CpG sites in general (29.7% and 4.8% of mappable probes, corresponding to 1.5- and 2.6-fold enrichments, respectively; Additional file 5). These correspond to cases where the impact of genetic variation is strong enough that classes of methylation levels are clearly distinct.

**Figure 5:**
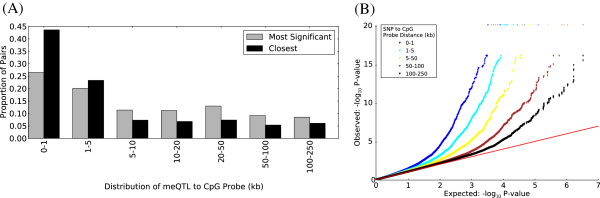
**mQTLs are preferentially close to CpG sites. (A)** Distribution of the mQTL to CpG probe distances for all correlated SNP-CpG pairs at 5% FDR. For each CpG probe, when more than one SNP is significantly correlated, a single one is retained as having either the most significant correlation (gray bars) or being located closest to the CpG probe (black bars). **(B)** Quantile-quantile plot of SNP/CpG probe Spearman’s rho *P*-values, grouped by pairwise distances. For each CpG probe included in the mQTL analysis, the most strongly correlated SNP within 250 kb was identified and the *P*-value obtained included in the set of *P*-values to be plotted for the distance bin in question. All SNPs in linkage disequilibrium with the selected SNP (R^2^ > 0.8) were removed, and the next most strongly correlated SNP was taken, until all SNPs within the range of the CpG probe in question were considered. The number of significant mQTLs decays with distance, but is still more than expected by chance at distances greater than 100 kb.

The majority (67%) of mappable CpG probes have a significant mQTL within 5 kb but in 6% of cases the closest significant mQTL lies more than 100 kb away (Figure 5A). Despite their relative rarity, these distal regulators of methylation appear genuine, since even at these larger distances, such pairs are seen much more often than expected by chance (Figure 5B).

### Linking gene expression and genetic variation (eQTLs)

**Figure 6:**
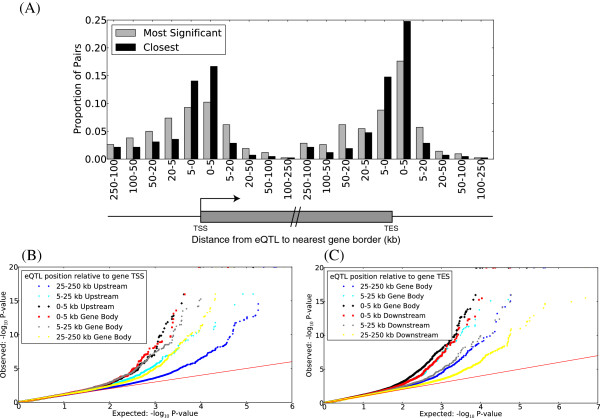
**eQTLs are concentrated near the transcription start and end sites of genes. (A)** Distribution of the distance between eQTLs and the closest of the boundaries (TSS or TES) of the gene whose expression they correlated with, for all pairs at 5% FDR. When a gene’s expression correlates significantly with more than one SNP, a single SNP is retained as having either the set of genotypes with the most significant correlation (gray bars) or being the most proximal to one of the two gene boundaries (TSS or TES). **(B**,**C)** Quantile-quantile plot of SNP/gene *P*-values, grouped by distances from the SNP to TSS **(B)** and TES **(C)**. Selection of *P*-values to be plotted followed a similar procedure to that in Figure 4B, with all SNPs located up to 250 kb on either side of the gene boundaries or within the gene body included for consideration.

We sought eQTLs within 250 kb of each gene with variable expression (absolute value Spearman’s rho >0.537, *P*-value <1.4 × 10^-5^, corresponding to a 5% FDR; see Materials and methods). Such eQTLs were found for 420 (4.4%) genes and involved 9,674 SNPs (Additional file 11). This is comparable to previous reports from Veyrieras *et al.*[24] (6.5% of genes mapping to an eQTL in LCLs, with a larger sample size of 210), but larger than the 2 to 3% seen by Stranger *et al.*[25] in four different HapMap populations. Consistent with previous reports [25], genes with eQTLs were not enriched for any specific GO annotations. As previously reported [24], eQTLs are most strongly over-represented near the TSS and transcription end site (TES) of genes, with a stronger enrichment within the gene body than outside (Figure 6).

**Figure 7:**
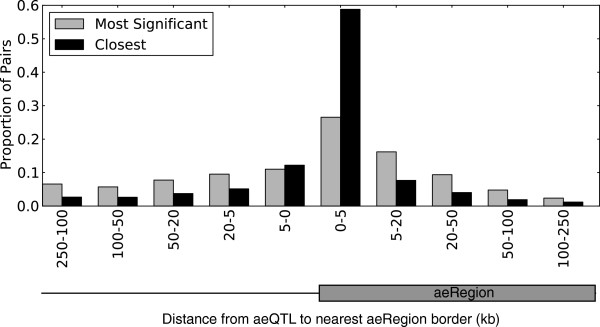
**aeQTLs are concentrated near boundaries of aeRegions.** Distribution of the distance between aeRegion boundary and the SNP they correlate with (5% FDR). When an aeRegion’s allelic expression correlates significantly with more than one SNP, a single SNP is retained as having either the set of genotypes with the most significant correlation (gray bars) or being the most proximal to one of the two aeRegion boundaries (black bars).

These eQTL data were complemented with the mapping of allelic expression ratios in aeRegions to candidate regulatory allelic expression quantitative trait loci (aeQTLs) within 250 kb (Spearman rho >0.452, *P*-value = 0.00029, corresponding to a 5% FDR; see Materials and methods). A total of 95,949 aeQTL-aeRegion pairs were obtained (Additional file 12), involving a total of 2,360 (or 71%) aeRegions and 89,874 candidate aeQTLs (many of which being in linkage disequilibrium with each other). These mappable aeRegions had a significant overlap with 1,452 annotated genes, three times more than the number of genes for which eQTLs were detected. We found 127 genes in both sets, corresponding to a 2.05-fold enrichment. Slightly larger overlap (2.92-fold enrichment) was observed in terms of the SNPs these genes mapped to. This significant but imperfect overlap by two methods is explained by multiple assay-specific factors: aeRegions are dependent on the presence of informative SNPs, are largely driven by primary transcript variation (intronic expressed SNPs) and in general allow for greater statistical power in terms of detecting statistically significant correlated SNPs [26] whereas eQTL mapping (conducted on Illumina expression arrays) assesses both transcriptional and post-transcriptional variation and is skewed towards measuring exon-specific variation [27]. Consequently, these methods can be used to complementarily capture different compartments of expression variation. Roughly 70% of mappable aeRegions have at least one candidate aeQTL within 5 kb of one of their boundaries (Figure 7), which is comparable to results seen using eQTL analysis with known genes.

### Linking gene expression to DNA methylation

We identified genes whose expression levels correlated with methylation levels of high-variance CpG probes located within their body or 250 kb on either end (absolute value Spearman’s rho >0.506, *P*-value <5.132 × 10^-5^, resulting in an FDR of 5%; see Materials and methods). This resulted in the identification of 587 genes with correlation to at least one of 1,793 CpG probes (Additional file 13). Extremely variable CpG sites are strongly over-represented amongst sites correlated with gene expression (Figure 4), and correlated CpG sites are 1.6-fold and 3.2-fold enriched, respectively, for bimodal and trimodal sites relative to the set of highly variable CpG sites.

Remarkably, methylation-correlated genes are far from representing an unbiased sample of the genome, with 78 (13%) of them being known transcription factors (GO enrichment *P*-value = 8.23 × 10^-16^) and 145 (24%) involved in multicellular organismal development (GO enrichment *P*-value = 6.1 × 10^-22^) (Additional file 6, worksheet 2). These include a number of genes from each of the four HOX clusters, together with several other key regulators of development and cellular differentiation such as EN1, HAND2, TBX1, TBX2, TBX3, TBX5, and TBX15.

**Figure 8:**
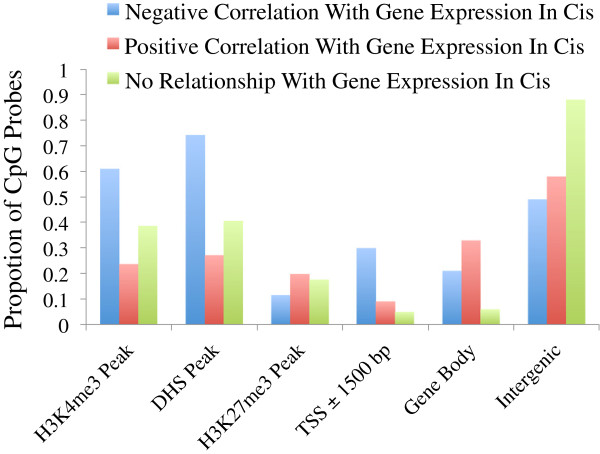
**CpG sites where methylation positively or negatively correlates with expression differ with respect to chromatin marks.** Proportion of CpG probes having various chromatin marks in at least one of five ENCODE fibroblast cell lines or located at various positions with respect to genes, with CpG probes grouped into three categories based on the type of correlation seen with an adjacent gene expression values.

**Figure 9:**
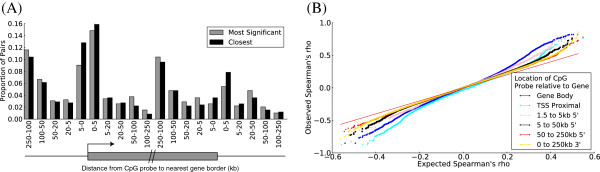
**Positive and negative methylation/expression correlations are seen at all positions with respect to the gene. (A)** Distribution of the distance between expression-correlated CpGs and the closest of the boundaries (TSS or TES) of the gene whose expression they correlated with, for all pairs at 5% FDR. When a gene's expression correlates significantly with more than one CpG site, it is retained as having either the set of methylation beta values with the most significant correlation (gray bars) or being the most proximal to one of the two gene boundaries (TSS or TES) (black bars). **(B)** Quantile-quantile plot of methylation/expression rank based correlation (Spearman’s rho), grouped by distances from the SNP to gene boundaries.

We sought to further characterize the CpG sites having methylation-expression correlations. Although about a quarter of methylation correlated genes had their closest correlated probe located within 1.5 kb of the TSS and 30% in their gene body, more than a third showed only correlation with distal intergenic probes (Figure 8). Since highly expressed genes have on average low DNA methylation near the TSS and higher DNA methylation at the gene body (Figure 3A), one might expect to see negative methylation-expression correlations for CpG probes located near a gene’s TSS and positive correlations for CpG probes located in its body. However, this is only partially verified, with one-third of the former type of pairs showing a positive correlation and nearly half of the latter showing a negative correlation. Overall, strong enrichments were seen for both negatively and positively correlated probes in both the gene body and TSS region, compared to other regions 3′ or more than 5 kb 5′ of the gene (Figure 9).

In order to find genomic features that may help distinguish CpG probes that correlate positively and negatively with gene expression, we turned to DHS and histone modification data obtained by the ENCODE consortium [8], considering data from five human fibroblast cell lines. Though these cell lines were not derived from the same donors as used in this study, we found in general that they allowed a clear separation between the two types of CpG probes (Figure 9). CpG probes where methylation levels correlated negatively with gene expression are for the most part located in regions with marks of regulatory activity (H3K4me3 or DHS): marks that are less frequent among CpG probes that show no correlation with expression and even less frequent among those that show a positive correlation. In contrast, positively correlated probes were slightly more often seen with the inactive gene-associated marker H3K27me3 when compared with negatively correlated probes.

**Figure 10:**
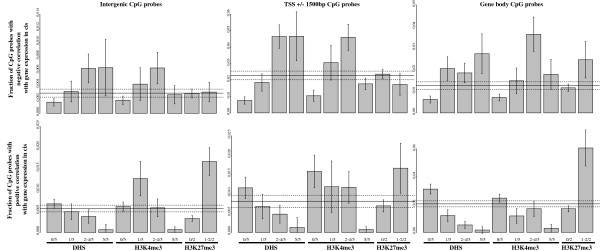
**The proportion of CpG sites where methylation correlates with expression depends on the site location, DHS and histone marks.** Proportion of CpG probes showing correlation with gene expression, ±95% confidence interval, for probes located in intergenic regions (left), within 1.5 kb of the TSS (middle), or within the gene body (right), and showing either negative (top row) and positive (bottom row) correlation, depending on the presence of DHS, H3K4me3 and H3K27me3. For DHS and H3K4me3 marks, the individual bars are based on the number (out of five) of ENCODE fibroblast cell lines that have the mark in question.

As illustrated in Figure 10, CpG sites in all types of genomic regions are more likely to be negatively correlated with gene expression if they are located in regions of DNase I HS in at least one of the five FB cell lines considered. A similar pattern was seen with the active transcription mark H3K4me3, with the notable difference that regions having this mark in all five fibroblast cell lines considered were under-represented for negatively correlated CpG marks, indicating perhaps that invariably active regions will also be subject to less consequential variability in terms of DNA methylation and expression. We also observe that regions containing H3K27me3 in at least one of the two fibroblast cell lines where this type of data was available are more likely to contain positively correlated CpG sites.

**Figure 11:**
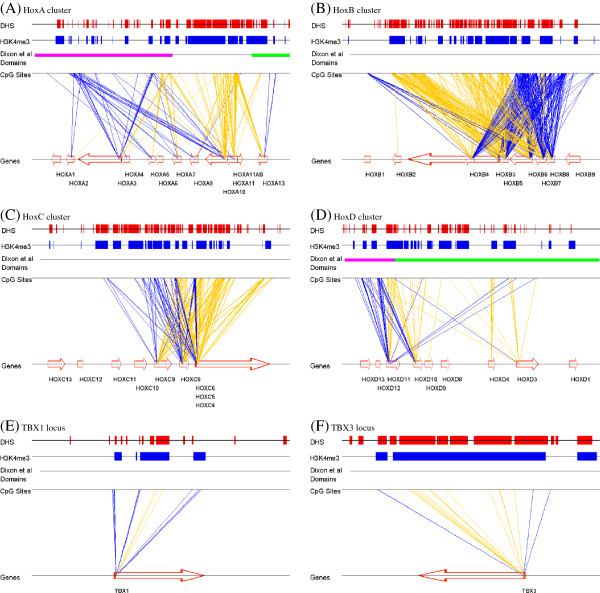
**Methylation-expression relationships in genomic context.** Schematic of significant methylation-expression relationships for **(A-D)** the four HOX clusters, and **(E**,**F)** genes *TBX1* and *TBX3*. Gold and blue lines link the TSS of the gene and the CpG probes correlated to that gene’s expression, with gold indicating negative correlation and blue indicating positive correlation. Red and blue blocks above indicate the presence of DHS or H3K4me3 marks in at least one of five ENCODE fibroblast cell lines. Where a domain boundary from Dixon *et al.*[28] was found, the domains are indicated with distinct colors.

In our samples, the four HOX clusters represent the densest centers of methylation-expression relationships in the genome. As seen in Figure 11A-D, each cluster is rich in both positive and negative methylation-expression correlations, involving CpG sites both within genes and within intergenic regions, with many but not all negatively correlated sites lying in regions marked by H3k4me3 and/or DHS. Also of interest in HOXA and HOXD are the topological domains obtained from a recent Hi-C study in IMR-90 cell lines [28]. In HOXD, a 40 kb region representing a boundary between the two domains contains the majority of CpG sites that have negative correlation with expression, whereas the boundary between two domains in HOXA also roughly delimits the positively and negatively CpG sites in this gene cluster. TBX1 and TBX3 represent other developmentally significant transcription factors having both positively and negatively correlated probes, whereas the latter largely coincide with DHS regions (Figure 11E,F).

### Overlap between mQTLs and eQTLs

**Figure 12:**
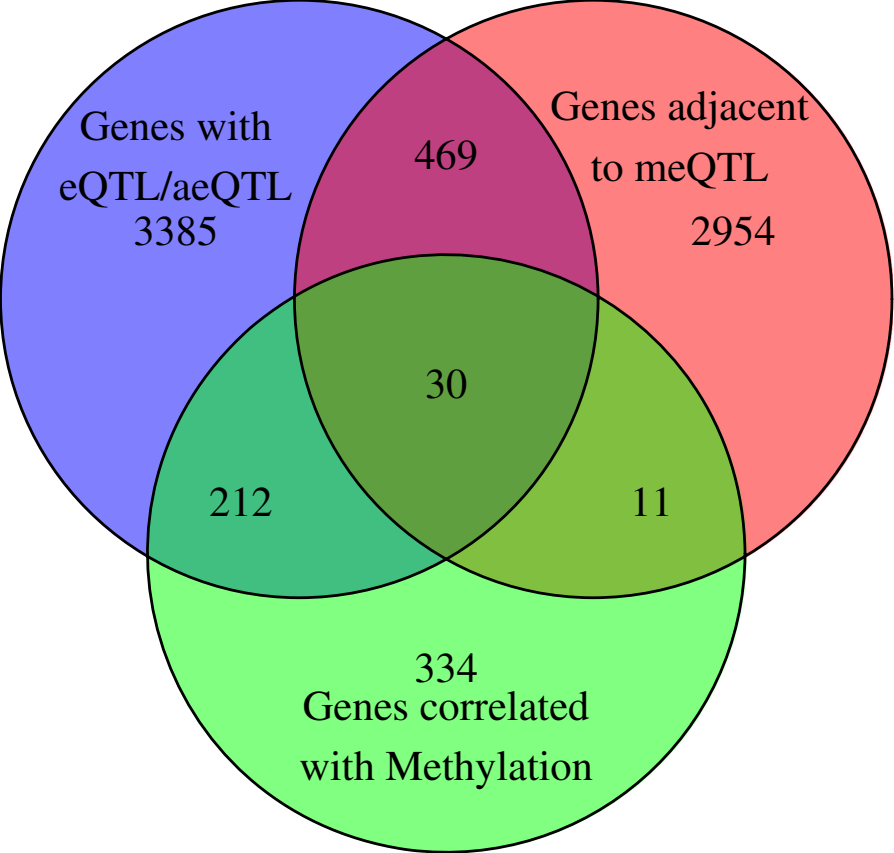
**Overlap of genes with an eQTL, genes with expression correlated with methylation, and genes adjacent to mQTLs.** Number of genes corresponding to various categories or relationships.

Three main types of relationships have so far been considered: methylation to sequence (mQTLs), expression to sequence (eQTLs and aeQTLs) and methylation to expression. To quantify the degree of overlap between the various relationships studied, we used genes, rather than CpG probes or SNPs, as the primary unit of interest. As seen in Figure 12, genes exhibiting two or three of the possible relationships form a relatively small but still non-negligible set. eQTLs and aeQTLs that were also mQTLs are termed in our report 'expression and methylation quantitative trait loci' (emQTLs), and correspond to a total of 52 eQTL-mappable genes and 234 aeQTL-mappable aeRegions, which together form the set of emQTL-mappable loci obtained in our analyses. When emQTL-mappable aeRegions are broken into annotated genes they overlap with, and merged with the list of emQTL-mappable genes obtained via combining eQTLs and mQTLs, we obtain a set of 242 emQTL mappable genes, plus 23 emQTL mappable aeRegions not overlapping with any annotated genes. Compared to a random selection of SNPs matched for minor allele frequency, we find 5.9 times more mQTLs are also emQTLs than expected by chance.

**Figure 13:**
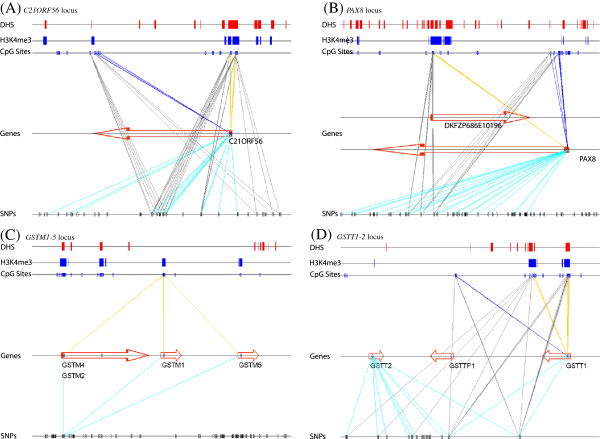
**emQTL relationships in genomic context.** Schematic of methylation-sequence-expression relationships in the loci surrounding the **(A)***C21ORF56*, **(B)***PAX8*, **(C)***GSTM1-GSTM5*, and **(D)***GSTT1-GSTT2* genes. Annotations are similar to those in Figure 13, with added grey and cyan lines indicating mQTL and eQTL relationships, respectively.

One example of an emQTL-mappable gene is *C21orf56* (Figure 13A), which had previously been reported as having mappable CpG probes near the TSS [11]. These probes overlap with DHS and H3K4me3 regions and are negatively correlated with expression. Also of note are positively correlated CpG probes located in the body of the gene, which are also mappable to a similar set of mQTLs.

Homeodomain transcription factor *PAX8*, transcription of which has been identified as an important biomarker in distinguishing various tumor types (reviewed in [29]), presented another particularly interesting case of overlap between the various types of relationships (Figure 13B), where CpG probes located near the gene’s TSS were unexpectedly positively correlated with the gene expression and those located in its body were negatively correlated. A possible explanation may involve putative uncharacterized transcript DKFZP686E10196, antisense to and located within *PAX8*, whose expression would be negatively correlated with the CpG methylation at sites near its TSS (but in the body of *PAX8*) but positively correlated probes in the body of the transcript (but near the TSS of *PAX8*). Indeed, RNA-seq data obtained from three individuals with differing genotypes in the *cis-*associated emQTLs suggest that the expression of *PAX8* and its antisense transcript are positively correlated, ruling out an interference between the two but instead hinting at a possible chromatin-linked role of DKFZP686E10196 activation in regulating *PAX8* transcription. (For a recent review of antisense regulation, see [30]).

Gene clusters of glutathione transferase families *GSTM* and *GSTT* also show multiple genes being mappable to similar sets of CpG probes and SNPs (Figure 13C,D), with active marks DHS and H3K4me3 located near negatively correlated CpG probes.

We estimated the proportion of gene expression variation that could be explained by either sequence variation alone or by a combination of sequence variation and DNA methylation, using a simple linear model and five-fold cross-validation (Materials and methods). For each gene, the five SNPs (within 250 kb) jointly explaining the largest portion of the expression variation on the training data were sequentially identified and regressed out. Independently of this we regressed out the five CpG sites explaining the largest portion of the expression variation. We found a total of 25.5% of gene expression variation to be explained by sequence variation, whereas methylation explained only 8.9% of expression variation. We applied a third model in which the top five SNPs were regressed out and then the top five CpGs were regressed from the residuals, finding in this case the variation explained by methylation dropped to 5.9%. This suggests that 5.9/8.9 = 66% of methylation-facilitated gene expression variation was independent of sequence variation. These figures are considerably higher than the 1.2% and 3.3% variation of expression explained, respectively, by DNA sequence and DNA methylation found by Li *et al.* in breast tumors [31], indicative perhaps of much greater variation of gene expression brought about by other factors in the tumor micro-environment.

## Discussion

We have analyzed the inter-individual variability of and relationships between one of the most comprehensive set of biomarkers in untransformed adult cells to date, including a more expansive assay for DNA methylation containing a large and diverse set of CpG dinucleotide probes; gene expression data; SNP data and allelic expression data, augmented with publicly available histone mark and DHS data from other cell cultures.

We chose primary skin fibroblasts as a model system. These comparatively easy to isolate and cultivate cells are a readily accessible source of patient material, and are in use as a model system for complex disease etiological studies (for example, Parkinson’s disease [32]). Epigenomes are tissue-specific, however, so the use of primary skin fibroblasts is limited to gaining insight into complex diseases of skin fibroblastic origin, or being a complementary tool with the requirement of additional studies of (the mostly more difficult to derive) primary patient tissue material. Of course, the limited sample size of this study reduced our ability to detect weak associations. However, complementing eQTL with mapping of AE significantly increases the sensitivity of our expression mapping [26], resulting in the discovery of many more expression/methylation QTLs than reported before.

Although most CpG sites with variable methylation seem unrelated to variation in gene expression, a non-negligible portion show significant correlations. Remarkably, the properties of these relationships appear quite complex, and the location of CpG probes with respect to the gene provides relatively little information about the sign of the correlation. Instead, chromatin states, particularly those that are representative of active chromatin and transcribed regions (DHS and H3K4me3) were more strongly indicative of negative correlation. Using the publicly available ENCODE data, we found in general that negatively correlated probes most strongly overlapped with regions of constitutive DHS but variable H3K4me3 among the five fibroblast cell lines considered, whereas positively correlated probes most strongly overlapped with an indicator of inactive transcription, H3K27me3. Work published in the ENCODE paper on DHS [8] indicated an inverse correlation of DNA methylation and DHS, and the authors provided evidence that DNA methylation was excluded as a consequence of open chromatin, rather than DNA methylation preventing this opening from occurring. H3K4me3 was also previously found to be inversely correlated with DNA methylation [9], with evidence for causality pointing in both directions. We have found further signs of intriguing links between all of these marks, and hope for experiments in the future more actively measuring these marks within the same cell lines to give better clues as to causality and to establish the constitutive and variable marks included in methylation-expression relationships.

Whether the associations between gene expression and methylation truly reflect variation in tissues or other differences acquired after sample collection is an important and challenging question. One possible source of post-sample collection variation is differences in cell proliferation rates. However, we have found that cell proliferation variation explained only 8% of the variance in methylation levels of expression-correlated CpG sites, and 13% of the variance in expression levels of methylation-correlated genes. Among mappable CpG sites and genes, the proportion of variance explained was negligible (0.7% and 5%, respectively).

Relatively high overlap was seen with results from previous studies in terms of the rare genes where both expression and methylation could be linked to genetic variation (emQTLs). In particular, *C21orf56*, a gene for which we find many emQTLs in fibroblasts*,* also exhibits the same property in whole blood [15] and LCLs [11]. Several other genes having emQTLs in whole blood [15] (*GSTM3*, *NAPRT1*, *SPG7* and *WBSCR27*) were also identified in our assay, indicating that genetic variation leading to both methylation and expression variation at the same locus is a relatively rare but reproducible phenomenon, the mechanism and implication of which merit further investigation. We report a total of 260 annotated genes or aeRegions that, to our knowledge, have not been previously reported as having emQTLs, including 23 aeRegions having no overlap to annotated genes. We attribute these discoveries to the usage of AE assays as well as a gene expression microarray experiments, together with use of the relatively recently developed Illumina Infinium HumanMethylation450 platform, interrogating methylation at a larger and more diverse set of CpG sites compared to most previous studies. As the effect of methylation on gene expression can in some cases involve cell-specific *trans*-acting factors [33], additional emQTLs could be found if we were to extend our analyses to additional cells or tissues. Future studies with larger sample sizes, investigating more diverse sets of cell types and utilizing platforms with even more comprehensive coverage of CpG sites can only help to uncover a greater number and potentially more subtle cases of associated DNA methylation, gene expression and DNA sequence variation.

Relationships between gene expression and DNA methylation in a population setting have not been investigated as extensively as sequence-expression or sequence-methylation relationships. However, previous high-throughput gene expression studies in fibroblasts have revealed intriguing results. In a landmark paper [34] assessing gene expression in skin fibroblasts derived from various anatomical sites, genes involved in a) extracellular matrix formation, b) cell signaling or fate determination, and c) cell migration signals were found to be expressed in a positional dependent fashion. Most notably of all, clustering of the samples based solely on the expression levels of 51 HOX genes recapitulated their site of origin. Koch *et al.*[35] strengthened these results by also finding positional-dependent DNA methylation at HOX loci in a set of skin fibroblast samples. In the present study, fibroblast samples drawn from the same site but from different individuals show considerable DNA methylation variation in CpG sites proximal to all four HOX clusters, and a subset of HOX genes are amongst those with the closest expression-methylation ties in the genome. However, the HOX genes with correlations to methylation reported differ from those previously found to have position-dependent expression [34], indicating additional layers of complexity and additional factors affecting fibroblast HOX methylation/gene expression beyond position in the body. Parents from several of the trios showed similar HOX expression and methylation profiles, indicating perhaps an environmental rather than a genetic origin for these characteristic patterns. Although this was not discussed in their paper, the data reported by Gutierrez-Arcelus *et al.*[17] also indicated that all four HOX clusters, as well as *PAX8*, showed high levels of methylation/expression correlations in each of the three cell types they studied. Future studies taking into account more carefully the environment and background of unrelated, healthy individuals will be paramount in understanding more clearly the factors at play in DNA methylation and gene expression of these fascinating loci. Overall, the inter-individual variability in gene expression seen in this fibroblast dataset, and the relationship of this variability to DNA methylation shows intriguing parallels to results seen with positional gene expression and DNA methylation variability in fibroblasts.

Genetic and methylation variation jointly explain 31% of gene expression variation in our fibroblast samples. However, the mechanisms involved appear complex and diverse, with a close interplay with other epigenetic marks. Further studies assaying inter-individual variation in histone marks and chromatin accessibility, ideally in an allele-specific manner, may bring the context necessary to the interpretation of sequence and methylation variation.

## Conclusions

We report a comprehensive analysis of relationships between sequence variation, DNA methylation and gene expression in untransformed adult human fibroblast cells. Consistent with previous reports showing positional effects in fibroblast on HOX gene expression [34] and DNA methylation [35], we show inter-individual variation and correlation between DNA methylation and gene expression in fibroblast cells even when drawn from the same location in the body. CpG sites with positive and negative correlations to gene expression show distinctive patterns with respect to the histone marks and chromatin accessibility seen in their genomic region in other fibroblast cell lines. We find in general the most remarkable relationships found with these data to be those involving gene expression and DNA methylation in developmentally significant regions having little or no discernible involvement of DNA sequence variation.

## Materials and methods

### Description of cell lines and cell culture

Primary skin fibroblasts were obtained from Coriell (Camden, NJ, USA) and the McGill Cellbank (Montreal, QC, Canada). Cells were grown in alpha MEM Medium (SigmaAldrich, Oakville, ON, Canada) supplemented with 2 mmol/l L-glutamine, 100 U/ml penicillin, 100 mg/ml streptomycin, and 10% fetal bovine serum (SigmaAldrich) at 37°C with 5% CO_2_ to 70 to 80% confluence, then harvested and stored at -80°C until RNA and DNA were extracted.

### DNA and RNA extractions

Genomic DNA (gDNA) for SNP genotyping and DNA methylation analysis was extracted from cell lysates using the GenElute DNA Miniprep Kit (SigmaAldrich) and DNeasy Blood and Tissue Kit (QIAGEN, Valencia, CA, USA), respectively, according to manufacturer’s protocol. DNA concentrations were determined using the Quant-iT PicoGreen kit (Invitrogen, Burlington, ON, Canada). Total RNA was extracted from cell lysates using the RNeasy Mini Kit (QIAGEN) according to the manufacturer’s protocol, and treated with 6 U DNase I. RNA quality was confirmed to be high for all samples on the Agilent 2100 Bio-Analyzer (Agilent Technologies, Mississauga, ON, Canada), with an RNA integrity number (RIN) range of 8.1 to 10, and concentrations were determined using the Nanodrop ND-1000 (NanoDrop Technologies, Wilmington, DE, USA).

### 450 K methylation array

gDNA (500 ng) was used for bisulfite conversion employing the EZ DNA Methylation Kit (Zymo Research, Irvine, CA, USA), according to the manufacturer’s protocols. The modified gDNA was processed as described in the Infinium Assay Methylation Protocol Guide Rev. C (November 2010), and analyzed on Infinium HumanMethylation450 BeadChips (Illumina; refer to [36] for more details), measuring DNA methylation at single CpG site resolution based on genotyping of C/U polymorphisms. We excluded probes with ≥90% sequence similarity to multiple genomic locations, probes with sequence variants in the probe-binding region and probes located on sex chromosomes, leaving 392,904 probes for further analyses. For removal of variant-containing probes HapMap (release 28, 30 CEU trios) annotated variants were imputed with 1000 Genomes project variants (pilot), and probes mapping more than one variant were removed. As a measure of methylation we chose the beta value, which theoretically ranges from 0, indicating no methylation at any allele, to 1.0 for complete methylation of both alleles.

Beta values of CpG probes were quantile normalized separately for type I and type II probes, with the reference distribution being the distribution of average per-probe beta values. Surrogate variable analysis [19] was carried out using the sva package in Bioconductor [37] and identified no hidden variables responsible for variation in data. Furthermore, following a methodology similar to that of Bell *et al.*[11], residuals obtained after regressing out up to five principal components were mapped to candidate mQTLs, and in none of the cases were a larger number of mQTLs or mQTL-mappable CpG probes obtained than with simply using quantile-normalized beta values; therefore, quantile normalized beta values were used throughout for further correlation analyses.

### Cell proliferation effects on expression and methylation

DNA concentrations from 8 individuals were used to obtain a set of 42 developmentally significant genes whose expression strongly (R > 0.75) correlates with DNA concentration. The first principal component of expression levels for the set of these 42 genes was obtained and used as a vector estimating the level of cell proliferation effects in the full set of individuals. For each methylation probe correlated either to gene expression or sequence variation, we carried out linear regression with the probe’s beta values and the cell proliferation vector. The variance of the residuals was compared with the variance of the original methylation probe, and done so cumulatively across probes to obtain the total variation in methylation of correlated probes explained by cell proliferation effects (with separate categories for CpG sites correlated to DNA sequence and gene expression). The process was repeated with expression probes found to be correlated with eQTLs and/or methylation of adjacent CpG sites to obtain an estimation of the total variation in expression of correlated genes explained by cell proliferation effects.

### Whole genome bisulfite sequencing

Whole genome bisulfite sequencing was carried out for cell lines GM02316, GM02317, GM02456, and GM02555 as described [38] with the modification that bisulfite conversion was carried out with the EZ DNA Methylation Kit (Zymo Research) according to the manufacturer’s protocol. We performed 100 bp paired-end sequencing on the Illumina Hiseq 2000 system; sequencing details are given in Additional file 13. Reads were mapped to the bisulfite converted reference genome using BWA and processed as described by Johnson *et al.*[39].

### Allelic expression measurement

AE measurement was carried out as described previously [22]. In short, approximately 200 ng gDNA and 50 to 300 ng double-stranded cDNA were genotyped in parallel on Illumina Infinium HumanOmni1-Quad, or HumanOmni2.5-Quad microarrays. The cDNA synthesis protocol was applied on heteronuclear RNA, allowing the measure of unspliced primary transcripts. For cDNA synthesis approximately 150 μg of total RNA was enriched using the MicroPoly(A)Purist protocol (Ambion Inc., Streetsville, ON, Canada). First strand cDNA synthesis was carried out on 1 μg poly(A)-enriched RNA using random hexamers, and second strand cDNA synthesis was performed using the Superscript Double-Stranded cDNA Synthesis Kit (Invitrogen). Data were filtered removing non-expressed SNPs and SNPs where cDNA arrays were unable to discriminate between homozygous genotypes, and normalized to compensate observed intensity dependent shift in median beta values of cDNA versus gDNA. For filtered SNPs obtained in the assay, smoothed scores of allelic expression were assigned based upon an eight-state left-to-right hidden Markov model (LTOR-HMM) as described in [23]. Based upon tests in which a null distribution was simulated by permuting raw allelic expression ratios independently within each sample, and on smoothed AE scores obtained from the LTOR-HMM, a threshold of 0.2 in at least two samples was identified as identifying allelically expressed SNPs with an FDR of 5%. Consecutive aeSNPs (ae-SNPs) having a smoothed AE value of the same sign and above this threshold in at least two individuals were aggregated into regions of allelic expression (aeRegions) and the mean smoothed AE score was obtained and assigned independently for each individual, in each aeRegion.

### Genotyping

Imputation of HapMap genotypes and phasing of Infinium HumanOmni1 and HumanOmni2.5-derived genotyping data were done using Beagle [40]. The SNPs used in correlation analysis throughout this study to obtain eQTLs, aeQTLs, mQTLs and emQTLs are all based upon this same set of SNPs.

### Gene expression arrays and eQTL analysis

Gene expression levels for 58 of the 62 individuals were determined using the Illumina HumanRef-8 Expression BeadChip according to the manufacturer’s protocol, giving expression levels for 21,916 probes mapping to a total of 16,952 genes.

These expression values were quantile normalized, the genes filtered such that only those in the top 50% variance of expression were retained, and expression values of these genes correlated using Spearman’s correlation coefficient to all SNPs within the gene boundaries or up to 250 kb upstream of the TSS or downstream of the TES. Expression values of the top 50% variable genes were permuted and the correlation analysis repeated to obtain a null distribution of *P*-values, and a *P*-value of 1.4 × 10^-5^ was obtained as the cutoff yielding a 5% FDR.

### Identifying allelic expression aeQTLs

All HapMap (release 28) SNPs at a distance of ±250 kb flanking each aeRegion and having a minor allele frequency >10% were correlated using Spearman’s correlation coefficient to their respective aeRegion. For each aeRegion, allelic expression values were permuted amongst the samples and the regression repeated to obtain an overall null distribution used in determining the FDR of *P*-values. A *P*-value threshold of 0.0029 was set based upon an FDR of 5%.

### Identifying methylation quantitative trait loci

Only probes having variance across samples in the top 25% were kept for correlation analysis with SNPs. Spearman’s rho was calculated between the highest 25% variance probes and HapMap SNPs at a distance of ±250 kb flanking each CpG probe and having minor allele frequency >10%. For each variable CpG probe, the analysis was repeated with methylation values permuted across individuals, in order to obtain a *P*-value of 6 × 10^-6^ for an FDR cutoff of 5%.

### Methylation-expression correlation

The same set of top 25% variable methylation probes and top 50% variable genes in the Illumina HumanRef-8 Expression BeadChip were used, obtaining the Spearman correlation coefficient between any methylation probe located within the body of an annotated gene or up to 250 kb on either side. Expression levels for each gene were permuted across the samples and the same set of Spearman correlation coefficients obtained, in order to set the *P*-value cutoff of 5.132 × 10^-5^ for a 5% FDR.

A CpG probe was labeled as being in the 'TSS' a gene if it was ±1,500 bp from its TSS. It was labeled as 'body' if it was not located within 1,500 bp of any TSS but was within an annotated transcript. Finally, it was labeled as 'intergenic' if it was neither 'TSS' nor 'body'.

The percentage contribution of methylation and sequence variation to expression variation was assessed using five-fold cross-validation and step-wise feature selection. For the training subset (80% of individuals), a linear model with expression of a particular gene as the response variable and genotypes of SNPs in the neighborhood of that gene as explanatory variables was selected using the stepAIC function in R [41]; the model was then used to predict expression values in the testing subset (1/5) of individuals. The same procedure of training and predicting was used across all five folds, and the R^2^ between the expression values and the predicted expression values using the models was obtained as the percentage of expression variation explained by sequence variation. The same procedure was repeated with the residuals of the gene expression values from the sequence-expression model as response variables and methylation beta values of CpG probes in the neighborhood as explanatory variables, in order to obtain the percentage of expression variation explained by methylation variation.

### Gene Ontology term enrichment

Significantly overrepresented GO categories were obtained for variable CpG probes and genes correlated to DNA methylation using Fisher’s exact test via GOStat [42], using default parameters available on the web server.

In the case of enrichment for highly variable CpG sites, genes with at least one top 25% variable CpG site at a TSS ± 1,500 bp were used as the test set; the set of all autosomal genes overlapping with at least one Illumina 450 K CpG probe was used as the background set. In the case of methylation-expression correlation, the set of all genes whose expression correlated significantly at 5% FDR with methylation of at least one CpG site were used in the test set; the set of all genes containing at least one CpG site within 250 kb was used as the background set. *P*-values were calculated by the GOStat web server, whereas fold enrichment was determined by dividing the proportion of genes in the test set with a given GO term by the proportion of genes in the background set with the same GO term.

### Overlap with DNase I hypersensitivity and histone markers

Data were downloaded from the ENCODE Data Consortium Center at UCSC at [43] on 15 October 2012, namely UW Dnase I HS and UW Histone broad peak data for fibroblast cell lines: Ag04449, Ag04450, Bj, Hff and Hcfaa (only Ag04450 and Bj were available for H3k27me3). A genomic locus was defined as having a given mark if that mark was present in at least one of the three cell lines. For each variable gene, the sets of a) positively correlated methylation probes, b) negatively correlated methylation probes, and c) all probes in a 250 kb neighborhood were obtained. For each category, the average (across genes) proportion of probes overlapping each type of mark was determined.

### Overlap between mQTLs and aeQTLs

The set of all SNPs that are categorized as correlated both to gene expression (aeQTL having relationship to an aeRegion and/or an eQTL correlated to a gene in the Ref8 array) and to DNA methylation (mQTL) at an FDR threshold of 5% in both of the respective analyses described above are categorized as methylation-regulatory SNPs (emQTLs).

### Data access

Methylation, gene expression, and SNP genotyping data for this publication have been deposited in NCBI’s Gene Expression Omnibus and are accessible through GEO SuperSeries accession number GSE53261.

## Abbreviations

AE: allelic expression; aeQTL: allelic expression quantitative trait locus; aeSNP: allelic expression single nucleotide polymorphism; bp: base pair; DHS: DNase I hypersensitivity; emQTL: expression and methylation quantitative trait loci; eQTL: expression quantitative trait locus; FDR: false discovery rate; gDNA: genomic DNA; GO: Gene Ontology; H3K27me3: histone 3 lysine 27 tri-methylation; H3K4me3: histone 3 lysine 4 tri-methylation; LCL: lymphoblastoid cell line; LTOR-HMM: left-to-right hidden Markov model; mQTL: methylation quantitative trait locus; SNP: single-nucleotide polymorphism; TES: transcription end site; TSS: transcription start site.

## Competing interests

The authors declare that they have no competing interests.

## Authors’ contributions

TP, MB, SB and JW conceived of the study. JW, MB, TP and SB wrote the article. TP, SB, BG and TK performed DNA methylation, gene expression, allelic expression and SNP genotyping experiments. JW, MB, and SB performed the computational analyses. All authors have read and approved the manuscript for publication.

## Supplementary Material

Additional file 1 Replicability of beta values in samples GM02456. Click here for file

Additional file 2 **Distribution of methylation beta values in type I probes across the genome, partitioned by position relative to (A) CpG islands and (B) annotated genes.** Click here for file

Additional file 3 **Proportion of type I CpG probes falling in various types of genomics regions identified by ENCODE, partitioned by (A) CpG probe mean beta value and (B) percentile of beta value standard deviation.** All data types, except for 28-way conservation, are derived from broad peaks in BJ human foreskin fibroblast cells. Click here for file

Additional file 4 **Mean (A) and standard deviation (B) of type I CpG probes with respect to their position relative to transcription start sites (TSSs) of annotated genes.** Each green dot corresponds to a CpG probe, and the four lines show the running median for probes based on the quartile of the expression level (from RNA-seq in four individuals) of the gene they are associated with. Click here for file

Additional file 5 **Proportion of CpG sites determined to have various numbers of modes in the set of individuals in the present study.** Independently for each CpG site, we applied the kernel smoothing algorithm in R [44], obtaining a set of 100 bins corresponding to a smoothed distribution of beta values. We then counted the modes, with a mode corresponding to a local maximum in the smoothed distribution having a y-value of at least 1.2 times the average. Click here for file

Additional file 6 **Enrichment/depletion of Gene Ontology terms, obtained using GoStat **[42]**, for highly variable CpG probes (worksheet 1) and genes with expression correlated to DNA methylation.** Click here for file

Additional file 7 Overlap of aeRegions with annotated genes. Click here for file

Additional file 8 Set of aeRegions. Click here for file

Additional file 9 Significant mQTL-CpG probe pairs. Click here for file

Additional file 10 Whole genome bisulfite sequencing (WGBS) statistics. Click here for file

Additional file 11 Significant eQTL-Ref8 gene pairs. Click here for file

Additional file 12 Significant aeQTL-aeRegion gene pairs. Click here for file

Additional file 13 **Signficant CpG probe-Ref8 gene methylation-expression correlation pairs.** Click here for file
