# Supplementary figures and images for: The relationship between DNA methylation, genetic and expression inter-individual variation in untransformed human fibroblasts
Source: Genome Biol. 2014 Feb 20;15(2):R37. doi: 10.1186/gb-2014-15-2-r37 (PMC4053980; doi:10.1186/gb-2014-15-2-r37)

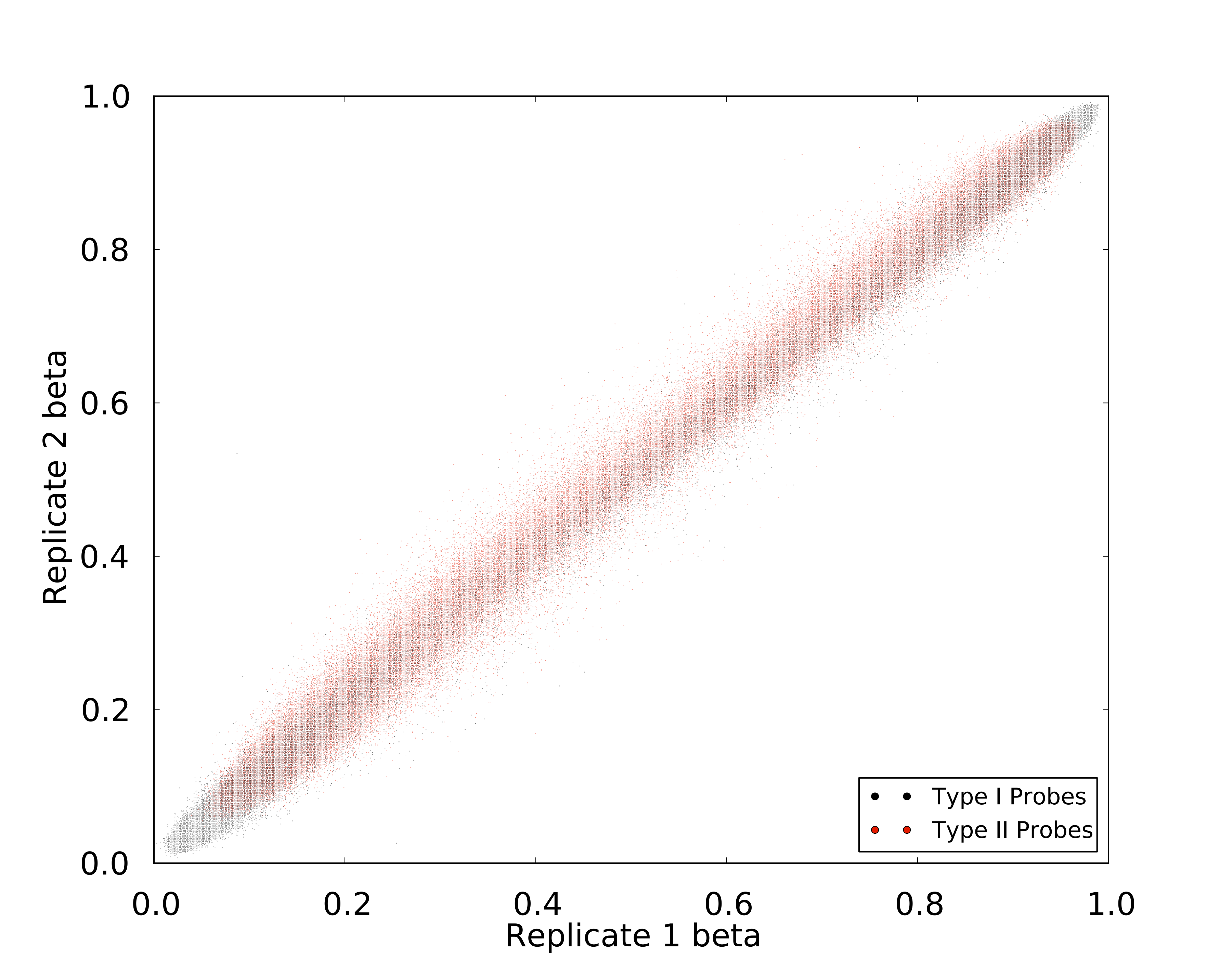

Supplement: Additional file 1 — Replicability of beta values in samples GM02456. [file gb-2014-15-2-r37-S1.tiff]

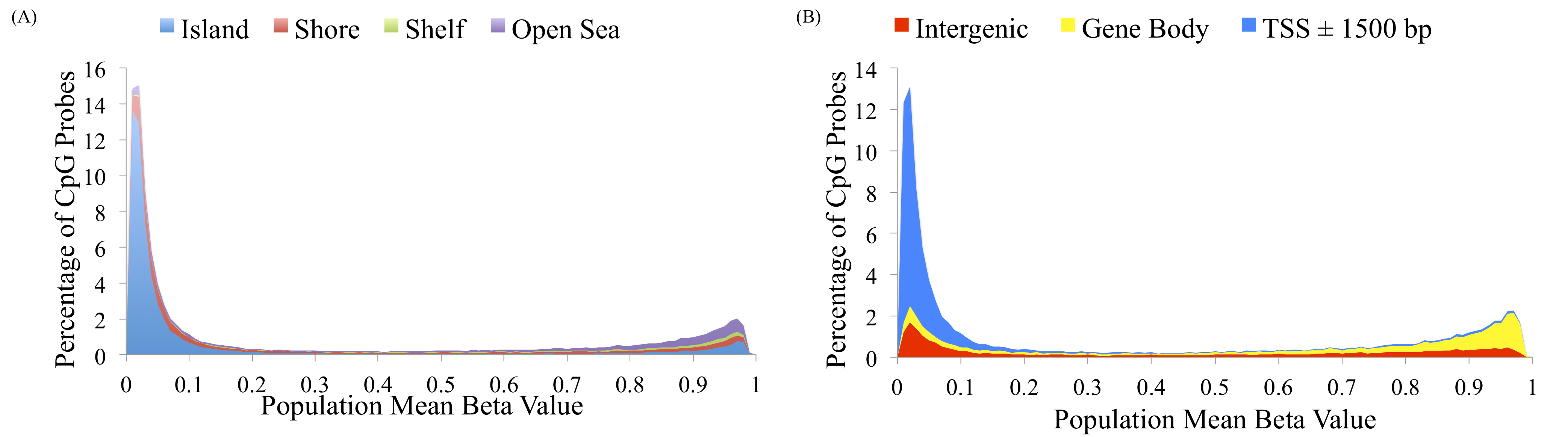

Supplement: Additional file 2 — Distribution of methylation beta values in type I probes across the genome, partitioned by position relative to (A) CpG islands and (B) annotated genes. [file gb-2014-15-2-r37-S2.tiff]

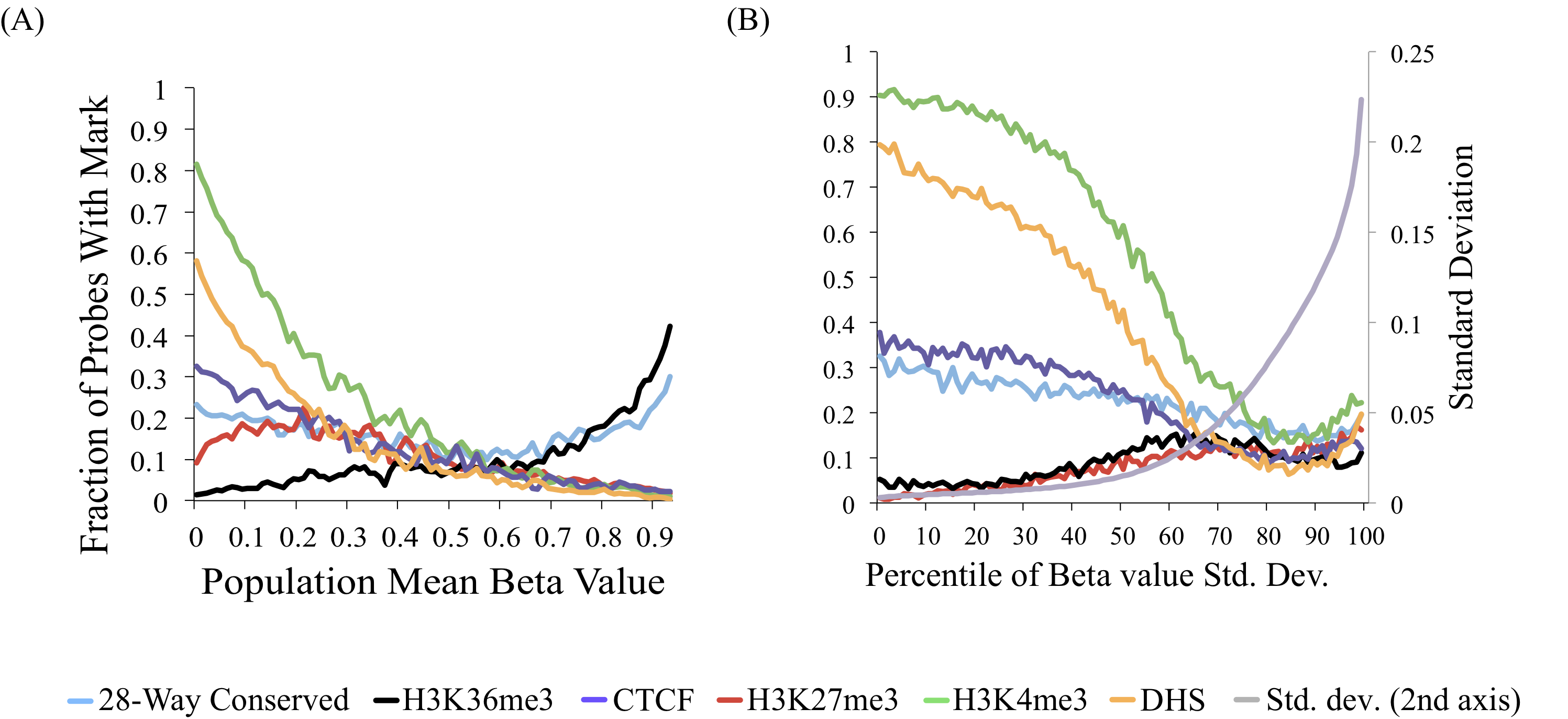

Supplement: Additional file 3 — Proportion of type I CpG probes falling in various types of genomics regions identified by ENCODE, partitioned by (A) CpG probe mean beta value and (B) percentile of beta value standard deviation. All data types, except for 28-way conservation, are derived from broad peaks in BJ human foreskin fibroblast cells. [file gb-2014-15-2-r37-S3.tiff]

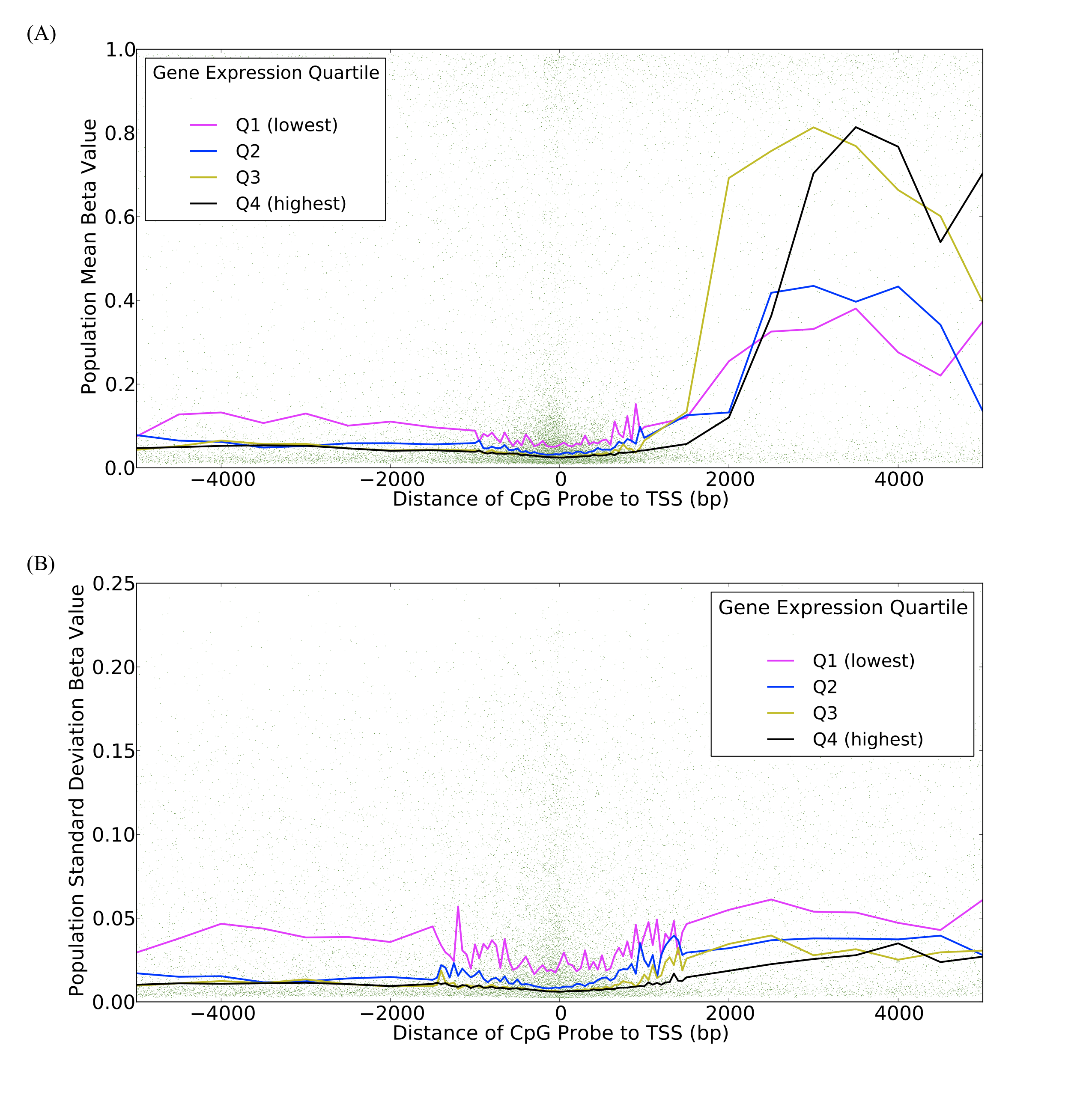

Supplement: Additional file 4 — Mean (A) and standard deviation (B) of type I CpG probes with respect to their position relative to transcription start sites (TSSs) of annotated genes. Each green dot corresponds to a CpG probe, and the four lines show the running median for probes based on the quartile of the expression level (from RNA-seq in four individuals) of the gene they are associated with. [file gb-2014-15-2-r37-S4.tiff]

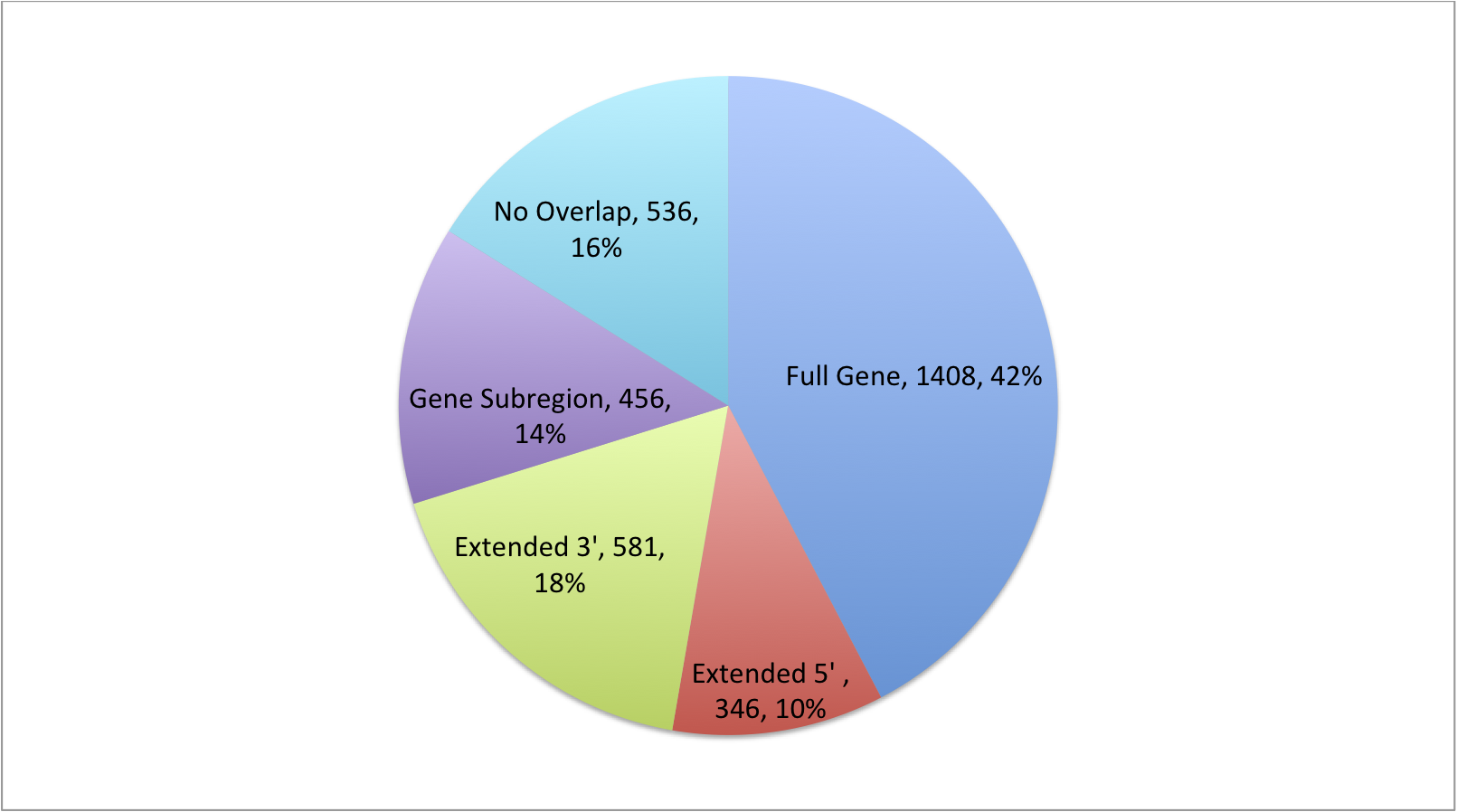

Supplement: Additional file 7 — Overlap of aeRegions with annotated genes. [file gb-2014-15-2-r37-S7.tiff]
